# Irradiation enhances susceptibility of tumor cells to the antitumor effects of TNF-α activated adipose derived mesenchymal stem cells in breast cancer model

**DOI:** 10.1038/srep28433

**Published:** 2016-06-22

**Authors:** Hemn Mohammadpour, Ali Akbar Pourfathollah, Mahin Nikougoftar Zarif, Amir Ali Shahbazfar

**Affiliations:** 1 Department of Immunology, Faculty of Medical Sciences, Tarbiat Modares University, Tehran, Iran; 2 Adult Stem Cell Research Center, College of Veterinary Medicine, Seoul National University, Seoul, Republic of Korea; 3 Blood Transfusion Research Center, High Institute for Research and Education in Transfusion Medicine, Tehran, Iran; 4 Department of Pathobiology, Faculty of Veterinary Medicine, University of Tabriz, Tabriz, Iran

## Abstract

Gene modified or cytokine activated mesenchymal stem cells (MSCs) have been used as a treatment in various types of cancer. Moreover, irradiation is usually applied as either a standard primary or adjuvant therapy. Here, we showed that the expression of TNF related apoptosis-inducing ligand (TRAIL) and Dickouf-3 (Dkk-3), the promising anticancer proteins, increased in murine adipose-derived mesenchymal stromal cells (AD-MSCs) following activation with TNF-α, resulting in the induction of apoptosis in cancer cells. Also, anticancer effects of TNF-α activated AD-MSCs were intensified with irradiation. *In vivo* results showed that TNF-α preactivated AD-MSCs combined with irradiation decreased tumor size and increased survival rate in tumor bearing mice. On the other hands, both TNF-α preactivated AD-MSCs with or without irradiation prevented metastasis in ling and liver, and increased apoptosis in tumor mass. Finally, flowcytometry assay demonstrated that naïve AD-MSCs combined with irradiation but not TNF-α activated MSCs with irradiation increased Treg population in lymph node and spleen. Altogether, obtained results suggest that TNF-α activated MSCs combined with irradiation therapy can serve as new strategy in breast cancer therapy.

Cancer is currently the second leading cause of death following heart disease and it is expected to be the first leading cause of death in the near future. Furthermore, Breast cancer is the third cause of death following lung and bronchus cancer in women^1^. Current treatment of breast cancer is based on combination therapy using radiotherapy, chemotherapy and cytotoxic molecules and antibodies targeting cancer cells^2^.

Radiotherapy as a crucial component in the management of breast cancer, implies high-energy to induce cell death in local tumors. There are a lot of evidences which indicate that the survival of patients is improved by radiotherapy through the reduction of local recurrence^3^. Radiotherapy is used either for treatment in a primary tumor or after surgery^4^. Radiotherapy also used as a component in combination therapy alongside with gene therapy^5^. On the other hands, it has been shown that radiotherapy modulates the tumor microenvironment by enhancing the recruitment of multipotent stromal cells (MSCs)^6^.

Multipotent stromal cells also known as mesenchymal stem cells are non-hematopoietic stem cells derived from diverse tissues such as bone marrow, umbilical cord and adipose tissue^7^. MSCs are considered for tumor therapy as a vehicle because they can preferentially migrate towards tumor site and incorporate in tumor stroma and this capability can be improved by radiotherapy^8^. In addition MSCs do not induce immune reaction in unrelated donor transplantation^9^.

Previous studies demonstrated that MSCs or gene modified MSCs could inhibit tumor growth^10^. More recently, some reports showed that antitumor effects of MSCs were improved upon activation with either TNF-α or IFN-γ through the up-regulation of TNF related apoptosis-inducing ligand (TRAIL) and Dickouf-3 (Dkk-3)^11^. Antitumor effects of TRAIL have been shown in many cancer cell lines without side effects on normal cells. TRAIL induces apoptosis in cancer cells through DR4 and DR5 receptors expressed in turmeric cells^12^. Because the soluble form of TRAIL exhibits short half-life and reduced efficacy, MSCs can be used as delivery vehicle. It has been shown that TRAIL over-expressed MSCs inhibited tumor growth^13^. Moreover, several reports have demonstrated that Dkk-3 plays important roles in tumor suppression and declined level of Dkk-3 is reported in diverse types of cancer. Dkk-3 suppresses tumor growth by the inhibition of Wnt signaling as an important signaling in tumorgenesis^14^.

Radiotherapy increased both expression of DR5 as receptor of TRAIL and MSCs migration toward tumor site^15^. In this study, we hypothesized that radiotherapy might increase the antitumor effects of cytokine activated AD-MSCs. Here, using *in vitro* and *in vivo* study, we investigated whether radiotherapy enhanced the therapeutic effect of TNF-α activated AD-MSCs by monitoring the tumor growth, survival rate, metastasis and regulatory T cell population in spleen and local lymph node.

## Results

### Proinflammatory cytokines increased expression of TRAIL, Dkk-3 and chemokines in AD-MSCs

**Figure 1:**
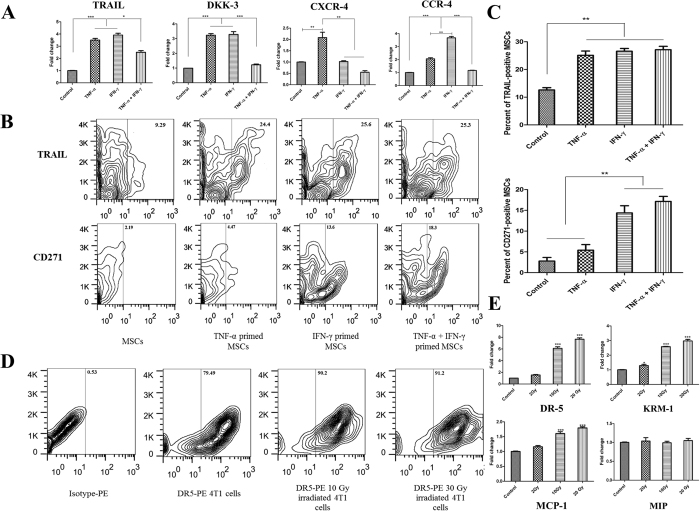
Preactivetion of MSCs with TNF-α increased the expression of TRAIL, DKK-3 and CXCR-4. (**A**) Real-time assay of TRAIL, DKK-3, CXCR-4 and CCR-4 in MSCs after activation with TNF-α, IFN-γ and TNF-α plus IFN-γ for 6 hrs. (**B**,**C**) flowcytometry assay of TRAIL and CD271 in AD-MSCs after activation with TNF-α, IFN-γ and TNF-α plus IFN-γ for 24 hrs. (**D**) Flowcytometry analysis of DR-5 expression in 4T1 cells after irradiation in different doses. (**E**) Enhanced expression of DR-5, Krm-1 and MCP-1 in 4T1 cells after irradiation confirmed by real-time PCR. All results are expressed by mean ± SD from at least three independent experiments analyzed by ANOVA with Tukey’s posthoc. *P < 0.05, **P < 0.01, ***P < 0.001.

At first step, the expression of TRAIL, DKK-3 and chemokines including CXCR-4 and CCR-4 were analyzed in AD-MSCs after activation with TNF-α, IFN-γ and TNF-α plus IFN-γ in different doses (data not shown). Finally, either TNF-α or IFN-γ was used at 20 ng/ml. In TNF-α plus IFN-γ group, 10 ng/ml of each cytokine was used. Our data showed that preactivation of AD-MSCs with either TNF-α or IFN-γ significantly upregulated the expression of TRAIL and DKK-3 compared to naïve AD-MSC and TNF-α plus IFN-γ activated MSCs (P < 0.001 and P < 0.05 respectively). In addition, CXCR4 and CCR4 expressions were significantly increased by the treatment of TNF-α or IFN-γ, respectively (P < 0.01 and P < 0.001 respectively) (Fig. 1A). At protein level, obtained data showed that expression level of TRAIL was significantly elevated in MSCs following preactivation with either TNF-α or IFN-γ and TNF-α plus IFN-γ (P < 0.01) (Fig. 1B,C). To select best candidate for AD-MSCs preactivation, we checked the immunosuppressive properties of AD-MSCs after MSCs activation either by TNF-α, IFN-γ or TNF-α plus IFN-γ. Our previous data indicated that TNF-α modulates the immunosuppressive effects of AD-MSCs on T cells and Dendritic cells compared to IFN-γ and IFN-γ plus TNF-α^16^. Moreover, we evaluated the expression of CD271 as a marker which displays the potent immunosuppressive AD-MSCs^17^. CD271 expression dramatically increased in AD-MSCs upon activation with IFN-γ and TNF-α plus IFN-γ but not TNF-α alone (P < 0.01) (Fig. 1B,C).

### Irradiation enhanced the expression of DR5 and Kremen1 in 4T1 cells

The expression of death receptor 5 (DR-5) and Kremen-1 (Krm-1) as main receptors of TRAIL and DKK-3 respectively, was analyzed after irradiation in 4T1 cells. The expression of DR-5 and Krm-1 at both mRNA and protein level was significantly up-regulated by irradiation compared to non-irradiated 4T1 cells (P < 0.001) (Fig. 1D,E). On the other hands, the expression of important chemokine receptors were evaluated and the expression of MCP-1 was noticeably increased after irradiation in 4T1 cells (P < 0.001) (Fig. 1E).

### Irradiation increased antitumor effects TNF-α activated AD-MSCs

**Figure 2:**
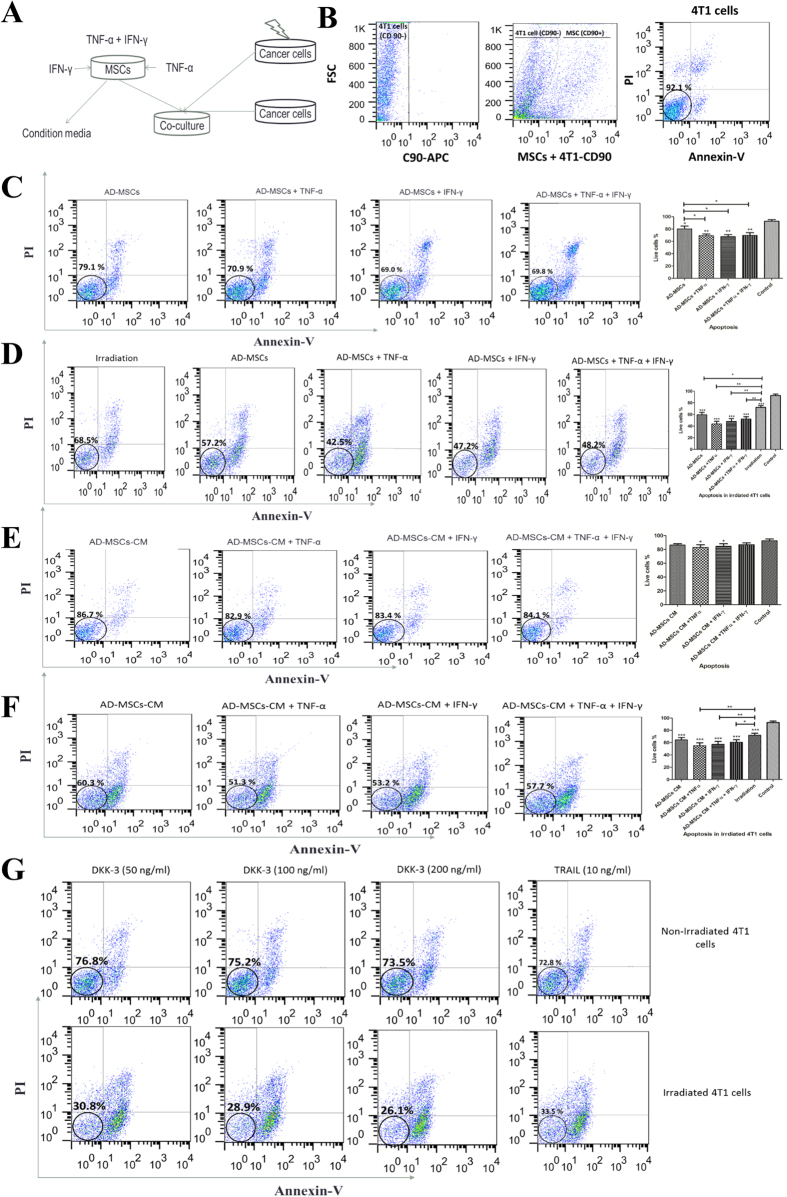
Irradiation increased antitumor effects of either cytokine preactivated AD-MSCs or cytokine preactivated AD-MSCs derived condition media. (**A**) Schematic diagram. (**B**) 4T1 cells were separated from MSCs by negative selection using CD90. Annexin-PI based flowcytometry analysis were used to check apoptosis and post apoptotic necrosis after co-culture for 24 hrs. Apoptosis assay was executed in (**C**) naïve or preactivated AD-MSCs and 4T1 cells co-culture, (**D**) naïve or preactivated AD-MSCs and irradiated 4T1 cells co-culture, (**E**) naïve or preactivated AD-MSCs derived condition media and 4T1 cells co-culture, (**F**) naïve or preactivated AD-MSCs derived condition media and irradiated 4T1 cells co-culture. rhDkk-3 and rhTRAIL were used as positive controls. Flowcytometry assay of induced apoptosis by (**G**) rhDKK-3 and rhTRAIL in 4T1 and irradiated 4T1 cells are presented. All results are expressed by mean ± SD from at least three independent experiments analyzed by ANOVA with Tukey’s posthoc. *P < 0.05, **P < 0.01, ***P < 0.001.

The apoptosis induction in naïve and irradiated 4T1 cells was analyzed following co-culture with naïve and activated AD-MSCs (Fig. 2A). We first isolated MSCs from 4T1 cells using CD90 as a specific marker for AD-MSCs (Fig. 2B). Our results showed that antitumor effects of naïve AD-MSCs were significantly enhanced following pre-activation with proinflammatory cytokines (P < 0.01; Fig. 2C) and this antitumor effects were dramatically increased after 4T1 cells irradiation (P < 0.001; Fig. 2D). As previously reported, MSCs derived condition media (MSCs-CM) can induce apoptosis and post apoptotic necrosis in cancer cells. Therefore, we next analyzed the percentage of live 4T1 cells after 24 hrs culture in the presence of 50% AD-MSCs-CM collected from preactivated AD-MSCs either by TNF-α, IFN-γ or TNF-α plus IFN-γ. Obtained results indicated that antitumor effects of AD-MSCs-CM were remarkably increased in either TNF-α or IFN-γ activated AD-MSCs but not in TNF-α plus IFN-γ group (P < 0.05; Fig. 2E) and the rate of apoptosis and post apoptotic necrosis on 4T1 cells significantly increased after irradiation (P < 0.01; Fig. 2F). On the other hands, irradiation also remarkably increased the apoptosis and post apoptosis necrosis induced by rhTRAIL and rhDkk-3 (P < 0.001; Fig. 2G).

### Irradiation combined with TNF-α activated MSCs decreased tumor size and improved survival of mice

**Figure 3:**
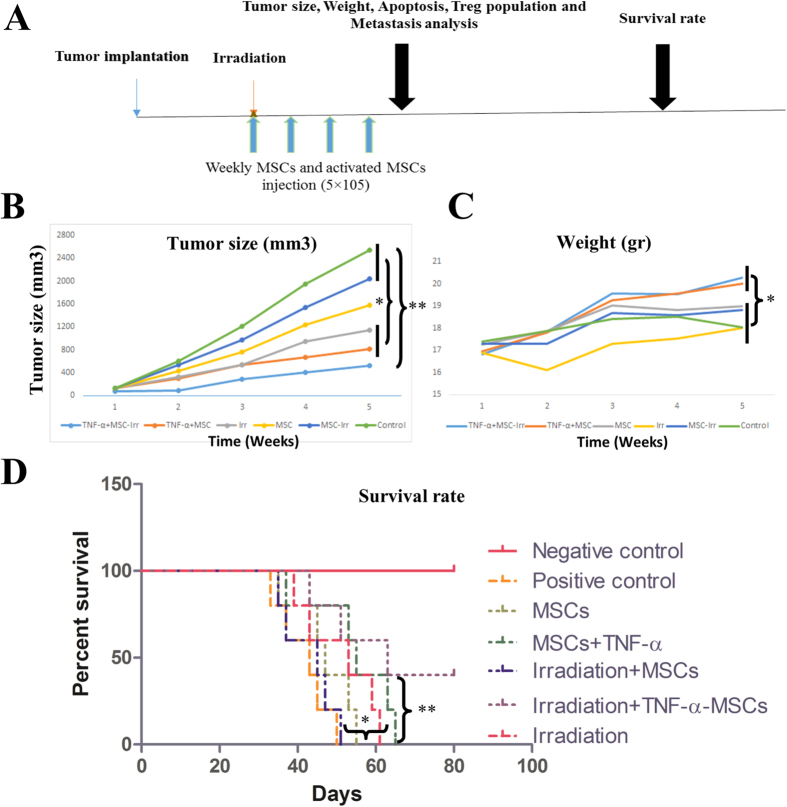
TNF-α activated MSCs combined with irradiation increased survival rate and weight of mice and decreased tumor size. (**A**) Schematic diagram of treatment strategy. Mice was inoculated with 1 × 10^6^ 4T1 cells and 2 weeks later, tumor mass (tumors were tangible) were irradiated with 6 Gy followed with 4 times weekly intratumoral MSCs injection for four weeks. (**B**) Tumor size and body weight (**C**) were assayed every 2 days with digital caliper and animal weight scale respectively. (**D**) Survival rate were assessed by a log-rank test based on the Kaplan–Meier method. All results are expressed by mean ± SD. *P < 0.05, **P < 0.01, ***P < 0.001.

Based on our current and previous studies, TNF-α was the best candidate for AD-MSCs preactivation. The schematic timeline of *in vivo* experiment is presented in Fig 3A. Tumor size in mice which received TNF-α preactivated AD-MSCs combined with irradiation was measured in comparison to the rest of mice (P < 0.01; Fig. 3B). TNF-α activated AD-MSCs or irradiation significantly decreased the tumor size compared to naïve MSCs or naïve MSCs with irradiation groups (P < 0.05; Fig. 3B). On the other hands, the body weight of mice as a possible indicator of quality of life, was measured. The body weight loss in TNF-α preactivated AD-MSCs plus irradiation and TNF-α preactivated AD-MSCs groups was significantly improved compared to the other groups (P < 0.05; Fig. 3C). Mice which received TNF-α preactivated AD-MSCs with irradiation treatment survived more than other groups (P < 0.01; Fig. 3D). Moreover, survival rate significantly increased in TNF-α activated AD-MSCs and irradiation groups compared to the groups of naïve MSCs with or without irradiation (P < 0.05; Fig. 3D).

### Combination therapy of irradiation with TNF-α activated MSCs prevented lung and liver metastasis and induced apoptosis in 4T1 cells

**Figure 4:**
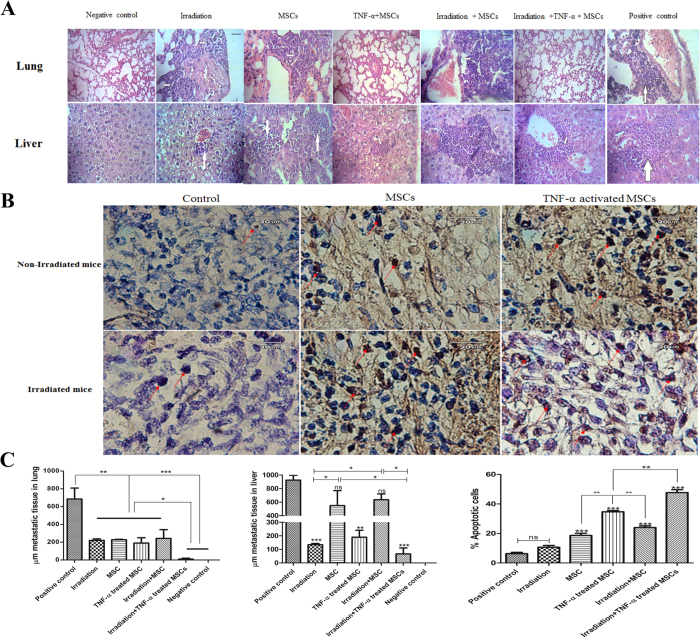
TNF-α activated MSCs combined with irradiation inhibited lung and liver metastasis and induced apoptosis in tumor mass. (**A)** Mice was inoculated with 1 × 10^6^ 4T1 cells and 2 weeks later, tumor mass (tumors were tangible) were irradiated with 6 Gy followed with 4 times weekly intratumoral MSCs injection for four weeks. One week after last injection, lung and live tissues were collected and the metastasis were checked by hematoxylin and eosin (H&E) staining. (**B**) One week after last injection induced apoptosis in tumor mass were analyzed by TUNNEL test. (**C**) The metastasis were quantified by measuring the presence of μmeter (μm) metastatic tissues in lung and liver. Apoptotic activities were quantified by measuring the percent of apoptotic bodies per 2,000 nuclei. All results are expressed by mean ± SD. *P < 0.05, **P < 0.01, ***P < 0.001.

The metastasis in liver and lung was assessed by measuring μm metastatic tissues in whole lung or liver organs. In all treatment groups, metastasis in lung was noticeably decreased compared to the control group (P < 0.01; Fig. 4A,C). Interestingly, combination therapy of TNF-α preactivated MSCs with irradiation dramatically abrogated metastasis in lung to a greater extent compared to other treatment groups (P < 0.05 and P < 0.001 respectively; Fig. 4A,C). Surprisingly, liver metastasis was remarkably reduced in irradiation, TNF-α activated AD-MSCs and TNF-α preactivated MSCs plus irradiation groups but not in naïve AD-MSCs and naïve AD-MSCs plus irradiation groups (Fig. 4A,C). We next analyzed the induced apoptosis in tumor tissues one week after last AD-MSC injection. The tumor in mice that received MSCs or preactivated MSCs contained more apoptotic cells compared to irradiation and control group (Fig. 4B,C). Additionally, both TNF-α preactivated AD-MSCs and TNF-α preactivated MSCs plus irradiation significantly induced apoptosis in the tumor cells compared to the naïve AD-MSCs and naïve MSCs plus irradiation respectively (P < 0.01). Moreover, apoptotic rate in tumor cells of mice which received TNF-α preactivated MSCs plus irradiation was significantly higher than that of mice with single treatment of TNF-α preactivated MSCs (P < 0.01; Fig. 4C).

### TNF-α activated MSCs with irradiation did not induce Treg cells in lymph node and spleen

**Figure 5:**
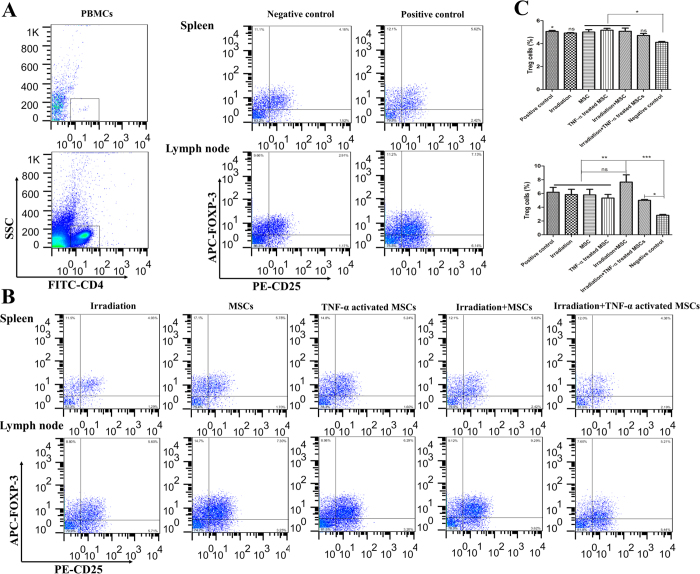
MSCs combined with irradiation but not TNF-α activated MSCs combined with irradiation increased Treg population in lymph node and spleen. (**A**) Regulatory T cells (Treg) population were analyzed by CD4+, CD25+ and FOXP-3+ in lymph node and spleen one week after last injection using flowcytometry. (**B**) Representative documentation of Treg cells by flowcytometry and (**C**) percentage of Treg cells in lymph node and spleen.

One of the important effects of MSCs is Treg induction in the tumor environment. Therefore, we analyzed Treg populations in spleen and local lymph node marked by detecting CD4^+^, CD25^+^ and FOXP-3^+^ cells (Fig. 5A). Tumor generation in mice significantly increased Treg population in both spleen and lymph node compared to healthy mice (Fig. 5A). Interestingly, Treg percentage in spleen was significantly increased in mice which received either MSCs, TNF-α preactivated MSCs or MSCs plus irradiation but not in irradiation and TNF-α preactivated MSCs plus irradiation groups (Fig. 5B,C upper panel). Furthermore, naïve AD-MSCs plus irradiation dramatically induced Treg cells in local lymph nodes (P < 0.001; Fig. 5B,C lower panel). Treg population in TNF-α preactivated MSCs plus irradiation group was increased, but to a lower extent than that in naïve MSCs plus irradiation group (P < 0.05; 5 ± 0.5 vs 7.65 ± 1.5; Fig. 5B,C).

## Discussion

MSC based cancer therapy have been recently demonstrated in several studies. Previous researches indicated that MSC therapy may lead to either promotion or prevention of tumor growth depending on the route or time point of injection, and type of tumor models^18^,^19^. Regarding breast cancer, Leng *et al.* demonstrated that human umbilical cord-MSCs (hUC-MSCs) inhibited tumor angiogenesis and induced apoptosis in MDA-MB-231 based breast cancer model in Nude mice^20^. More recently, it has been shown that MSCs derived from iPSCs do not promote tumor growth^21^. Moreover, MSCs inserted with IL-2^22^, IL-12^23^, IL-21^24^, TRAIL^25^ and DKK-3^26^ genes have been used in wide range of cancer models with promising results. Conversely, it has been indicated that cancer stroma resident MSCs have increased tumor growth through the promotion of cancer metastasis, providing a carcinoma stem cell niche and cytokine networks^27^,^28^.

In the present study, we demonstrated that TNF-α activated MSCs combined with irradiation is more advantageous comparing with either irradiation or TNF-α activated MSCs alone. We used TNF-α activated MSCs instead TRAIL-expressing MSCs because, MSCs activation by TNF-α increased expression of TRAIL, DKK-3 and migratory protein in MSCs altogether, modulates immunosuppressive effects of MSCs and is easier to apply compared to gene insertion by delivery systems such as an adenoviral or lentiviral vectors. Obtained data showed that expression of TRAIL and Dkk-3 as main antitumor factors was increased in AD-MSCs following activation with TNF-α. Here, we focused on TRAIL and DKK-3 proteins, because previous studies showed that anticancer effects of MSCs are mainly related to these factors^13^,^29^. Also, Lee *et al.*, showed that siRNA inhibition of either TRAIL or DKK-3 strongly inhibit anticancer effects of MSCs in MDA cell line^11^. We further showed that irradiation enhanced susceptibility of 4T1 cells to activated AD-MSCs as well as MSC derived condition medium through the up-regulation of DR5 and Krm-1. We demonstrated that TRAIL and Dkk-3 efficiently induced apoptosis and post apoptosis necrosis in 4T1 cells, implying that 4T1 cells are sensitive to both TRAIL and Dkk-3. Moreover, irradiation increased 4T1 sensitivity to anticancer effects of TRAIL and Dkk-3 through upregulation of DR-5 and Krm-1 receptors. These data are in parallel with reported results by Kim *et al.*, demonstrating increased susceptibility of glioma cell line to anticancer effects of TRAIL-expressing MSCs. Also, they showed that migration capability of MSCs toward tumor site increased by irradiation through enhanced secretion of IL-8 by irradiated tumor cell^15^. Previous studies indicated that TRAIL is a potent anticancer protein and that its effects can be synergistically increased by chemotherapy and irradiation^15^,^30^. TRAIL induced apoptosis in cancer cells through DR4/DR5 in human and DR5 in mice as well as activating caspase pathway demonstrated in lung, breast, and glioblastoma cancer cells^31^. Moreover, some researches showed that apoptotic cell-derived RNA and DNA can activate MSCs and other immune cells through TLRs such as TLR-3 and provides a feed forward reaction, leading to the increase in TRAIL expression and subsequent tumor suppression^32^. On the other hands, there are several reports which showed that Dkk-3 implies antitumor effects through cell cycle inhibition via Wnt-β-catenin inhibition^33^, EGFR blocking^34^ and more recently, apoptosis induction^35^. Previous investigations reported that Wnt-β-catenin inhibition and EGFR blocking are potential treatment for cancer^36^,^37^. On the other hands, it has been shown that MSCs derived condition media inhibited cancer cells growth^38^. Condition media derived Dkk-3 induced apoptosis through mitochondrial and Fas death receptor pathways^35^. In addition, condition medium derived from MSCs down-regulated the expression of vascular endothelial growth factor partially by miR-16 and suppressed angiogenesis^39^.

In this study, we showed that tumor growth, survival rate and metastasis were not significantly altered by naïve (without cytokine activation) AD-MSCs injection. However, TNF-α preactivated AD-MSCs significantly inhibited tumor growth, metastasis and improved survival rate in tumor bearing mice and this antitumor effects were remarkably intensified by single dose irradiation one day prior to initial administration of cells. In the present study, AD-MSCs were injected by intratumoral route, because previous results showed that intratumoral injection of MSCs exhibited potent anticancer effects with more maintenance and stability in comparison to other injection routes^40^. Furthermore, intratumoral injection provided enough numbers of MSCs in tumor site. Also, it has been shown that MSCs are mostly trapped in lung when injected intravenously^41^. We used irradiation as an adjuvant therapy that have increased antitumor effects of modified MSCs reported by several reports. Balyasnika *et al.*, showed TRAIL expressing MSCs in combination with irradiation promoted survival rate in brain tumor bearing mice^42^. More recently, it has been shown that irradiation enhanced curative effects of IL-12 expressing MSCs in murine metastatic hepatoma by inducing MSCs localization and increasing apoptotic activity^43^. Radiotherapy enhanced tumor cell sensitivity to TRAIL by inducing expression of DR-4/DR-5 in human or DR-5 in mice confirmed in wide range of tumor such as lung, colon, head and neck, prostate and breast cancer. Furthermore, it has been found that radiotherapy also induced stress pathways and death signaling in independent manner in cancer cells^44^.

Finally, it has been demonstrated that the number and activity of Treg increased by the soluble factors secreted from tumor associated MSCs, such as IL-10 and TGF-β^45^. Therefore, we here examined Treg population in spleen and lymph node following tumor inoculation. Interestingly, naïve MSCs but not TNF-α preactivated MSCs with irradiation induced Treg development in lymph node. This effect is possibly related to MSC recruitment from other tissues towards tumor mass. Because it has been previously shown that irradiation increased the migratory properties of MSCs by inducing the secretion of VEGF, tumor growth factor-β (TGF-β) and platelet-derived growth factor (PGEF) by tumor cells. Furthermore, enhanced level of TGF-β and PGEF in tumor cells can directly contribute to the development of Treg cells in tumor microenvironment^6^.In preactivated MSCs, it seems that high level of apoptosis induction decreased the tumor cell mass as a main source of soluble factor release which in turn recruit MSCs and induce Treg cells. Moreover, some studies showed that TNF-α modulated the immunosuppressive effects of MSCs^16^,^46^.

In conclusion, these data suggest that intratumoral delivery of TNF-α preactivated MSCs immediately after single dose irradiation could be a potential strategy for cancer treatment through the strongest antitumor effects as well as significant suppressive action on metastasis of breast cancer.

## Material and Methods

### Mice and tumor lines

Six to eight weeks old female Balb/c mice from Pasteur institute, Tehran, Iran were used according to institutional guidelines approved by Faculty of Medical Sciences, Tarbiat Modares University, Tehran, Iran. All mice were kept in approved animal facility with ad libitum access to food and water under 12 hrs dark and light. Breast adenocarcinoma (4T1) mouse cancer cells and mouse embryonic fibroblast (MEF) cell lines were provided from Pasteur institute, Tehran, Iran. Mouse adipose derived MSCs (AD-MSCs) were isolated and confirmed based on our previous study^16^,^47^. Mice were divided into 7 groups including healthy group (tumor negative group), tumor control, Single irradiation, MSCs, TNF-α activated MSCs, MSCs plus irradiation, TNF-activated MSCs plus irradiation.

### Reagents and antibodies

All used antibodies including PE conjugated TRAIL, PE conjugated CD271, FITC conjugated CD4, PE conjugated CD25, APC conjugated FOXP-3, PE conjugated DR-5 and APC conjugated CD90 were purchased from eBioscience company (San Diego, USA). The rhTRAIL and rhDKK-3 proteins were provided form PeproTech and Sino Biological companies, respectively (Seoul, South Korea and North Wales, USA, respectively).

### Real-time PCR

Real-time PCR procedure was executed based on the 1 μg/μl cDNA in all samples. Primer pairs for all considered genes are listed in Supplementary Table-1. The exact mRNA expression was normalized to the expression level of GAPDH.

### Irradiation

4T1 and MEF cells were irradiated with different doses including 5, 10 and 30 Gy provided by Gammacell 3000 Elan source. Irradiation of the animals was performed two weeks after tumor induction using a Co-60 unit (Imatron, Canada) as the gamma radiation source at a dose 6 Gy single. Tumor bearing mice were fixed using an acrylic chamber to avoid mice movement and precisely expose the tumors of the mice. The body of mice except tumor site was shielded using a specially designed lead apparatus.

### Cocultures of AD-MSCs and 4T1 Cells

AD-MSCs were cultured in low glucose Dulbecco’s Modified Eagle’s Medium (DMEM) supplemented with 10% fetal bovine serum (FBS), 1% penicillin/streptomycin at 37 °C with 5% CO_2_. 4T1 cancer cells were grown in Roswell Park Memorial Institute (RPMI 1640) with 10% FBS and 1% penicillin/streptomycin. AD-MSCs were activated by 20 ng/ml TNF-α 24 hrs before co-culture. After 24 hrs, AD-MSCs were twice washed by PBS and 1 × 10^5^ AD-MSCs were cocultured with naïve and irradiated 4T1 cells in ratio of 1:1 for 24 hrs. For condition medium (CM) preparation, after AD-MSCs activation by TNF-α, the medium was replaced and maintained for another 24 hrs. After 24 hrs, condition medium was collected and stored in −70 °C for further analysis. Naïve and irradiated 4T1 cells were cultured with 50% activated AD-MSCs derived CM and 50% normal culture medium for 24 hrs.

### Flow Cytometry

4T1 cancer cells were separated from AD-MSCs cells by negative selection using APC conjugated CD90 antibody. Apoptosis was evaluated using Annexin & PI apoptosis kit (eBioscience, San Diego, USA) in naïve and irradiated 4T1 cells according to the manufacture’s instruction. The regulatory T cells (Treg) population was analyzed by Treg kit based on the manufacture’s protocol in spleen and local lymph node.

### Breast cancer Model, AD-MSCs Infusion and Tissue Collection

Breast cancer model in Balb/c mice were induced by subcutaneous injection of 0.2 ml Phosphate buffered saline (PBS) containing 1 × 10^6^ 4T1 cells to the right hind limb of the mice. After two weeks, when the tumor size was tangible, mice were irradiated with 6 Gy. After one day, naïve and TNF-α activated AD-MSCs were weekly injected into tumor area for 4 weeks. One week after last injection, liver, lung and tumor tissues were collected for further analysis.

### Tumor size measurement

The tumor dimensions were measured every two days by using a digital caliper and the tumor volume was calculated with the following formula: tumor volume [mm3] = (length [mm]) × (width [mm]) 2 × 0.52.

### Lung and liver metastasis

Lung and liver tissues were fixed by 70% formalin and were stained with Hematoxylin and Eosin (H&E) dyes according to the previous studies^48^.

### TUNEL assay

Tissue were fixed with paraformaldehyde and embedded in paraffin. *In vivo* apoptosis were analyzed using *In Situ* Cell Death Detection Kit, POD (Roche, Mannheim, Germany) based on manufacture’s protocol. In each group, at least ten area were selected to detect the apoptosis. Apoptotic activities were quantified by measuring the percent of apoptotic bodies per 2,000 nuclei.

### Statistical analysis

Obtained results were analyzed by GraphPad Prism version 4.0 and SPSS 16.0. Two-tailed Student’s t-test and ANOVA with post-hoc Tukey’s Multiple Comparison tests were used for differences between two groups and more than two groups respectively. Kaplan- Meier test was used for survival and comparison of groups was determined by Log-rank (Mantel-Cox). P value <0.05 was considered as statistically significant.

## Additional Information

**How to cite this article**: Mohammadpour, H. *et al.* Irradiation enhances susceptibility of tumor cells to the antitumor effects of TNF-α activated adipose derived mesenchymal stem cells in breast cancer model. *Sci. Rep.* **6**, 28433; doi: 10.1038/srep28433 (2016).

## Supplementary Material

Supplementary Information
