# Supplementary material for: Irradiation enhances susceptibility of tumor cells to the antitumor effects of TNF-α activated adipose derived mesenchymal stem cells in breast cancer model
Source: Sci Rep. 2016 Jun 22;6:28433. doi: 10.1038/srep28433 (PMC4916474; doi:10.1038/srep28433)
Supplement: Supplementary Information [file srep28433-s1.doc]

**Irradiation enhances susceptibility of tumor cells to the antitumor effects of TNF-α activated adipose derived mesenchymal stem cells in breast cancer model**

Hemn Mohammadpour^1,2^, Ali Akbar Pourfathollah^1*^, Mahin Nikougoftar Zarif ^3^, Amir Ali Shahbazfar^4^

1. Department of Immunology, Faculty of Medical Sciences, Tarbiat Modares University, Tehran, Iran
2. Adult Stem Cell Research Center, College of Veterinary Medicine, Seoul National University, Seoul, Republic of Korea
3. Blood Transfusion Research Center, High Institute for Research and Education in Transfusion Medicine, Tehran, Iran
4. Department of Pathobiology, Faculty of Veterinary Medicine, University of Tabriz, Tabriz, Iran

***Corresponding author:** Ali Akbar Pourfathollah, Department of Immunology, Faculty of Medical Sciences, Tarbiat Modares University, Tehran, Iran. Postal Code: 14115-331. Tel: +98-21-82883874, Cell: +98-912-3548650 Email: [Pourfa@modares.ac.ir](mailto:Pourfa@modares.ac.ir)

Table 1: **Primers list used for Real-time PCR**

| Reverse 5 to 3 | Forward 5 to 3 | gene |  |
| --- | --- | --- | --- |
| AGACGGTTCCAGGAGTCAAAGG | AAAACGGCTTGGGCATCTTGGC | DR5 | 1 |
| TGAGAAGCAAGCTAGTCCAATTTT G | CAG GCT GTG TCT GTG GCT GT | TRAIL | 2 |
| GATGATATGGACAGCCTTACAC | AGC ATG ACG GAC AAG TAC C | CXCR4 | 3 |
| TTT CGG GTC AAT GCA CAC TTG | GAG AGC CAC ATC GCC AGA G | SDF-1 | 4 |
| GCTATCTTCCGCCAGGCATAT | AATCTGTTGTGGCTTCACCCA | CXCR1 | 5 |
| ACGAGCTAACAAAAGAAGGCCTT | GAAATTTCGCCATGGACTTCTC | CXCR2 | 6 |
| TTCAGGGTCAAGGCAAACTT | AGTGAACTGCGCTGTCAATG | MIP2 | 7 |
| CTCGCTCCTGGAAGATGGTG | GGTGAAGGTCGGTGTGAACG | GADPH | 8 |
| GACGGGGTTAAGGCAGCAGTGA | ATCGTGCACGCGGTATTCTCC | CCR4 | 9 |
| CAGAAGTGCTTGAGGTGGTTGTG | ATCCCAATGAGTAGGCTGGAGAGC | MCP-1 | 10 |
| ACCAAAGCTGCAGAAGTCTC | TCCCTTTCTGGCTAACAGGA | DKK3 | 11 |
| TCC ATC CCA GCA AAC TTG AAT C | AAC GAG ACT TTC CAG CAT CCG | KRM1 | 12 |
| CGT CCA AGG CAC CAT CTC TTT G | TGGGTTCCTACAGAA GTT ATGCG | KRM2 | 13 |
